# Associations between fruit and vegetable, and antioxidant nutrient intake and age-related macular degeneration by smoking status in elderly Korean men

**DOI:** 10.1186/s12937-017-0301-2

**Published:** 2017-12-04

**Authors:** Eun-kyung Kim, Hyesook Kim, Aswathy Vijayakumar, Oran Kwon, Namsoo Chang

**Affiliations:** Department of Nutritional Science and Food Management, Ewha Womans University, Seoul, 120-750 Republic of Korea

**Keywords:** Age-related macular degeneration, Fruit and vegetables, Antioxidants, Elderly male smokers, KNHANES

## Abstract

**Background:**

Age-related macular degeneration (AMD) is one of the major causes of irreversible blindness. The objective of this study was to determine whether there is any relationship between dietary intake of fruits and vegetables (F&V) and antioxidant nutrients including carotenoids and AMD according to smoking status in elderly men.

**Methods:**

We performed a cross-sectional analysis using nationally representative samples of elderly aged ≥ 65 years (*n* = 1414) from the Korea National Health and Nutrition Examination Survey (KNHANES, 2010–2012).

**Results:**

The current smokers consumed less food in total, and, in particular, less cereals/potatoes/sugar products, fruits and vegetables than the nonsmokers and former smokers (*p* < 0.05). Intake of energy, thiamin, vitamin C, vitamin A, and β-carotene were significantly lower in the current smokers than in the nonsmokers and the former smokers. For current smokers, the ORs of the highest tertile compared with the lowest tertile were 0.36 (95% CI: 0.14–0.96, p for trend = 0.0576) for F&V, 0.32 (95% CI: 0.12–0.85, p for trend = 0.0561) for vitamin C, 0.23 (95% CI: 0.08–0.67, p for trend = 0.0038) for α-carotene, 0.13 (95% CI: 0.04–0.46, p for trend = 0.0003) for β-carotene after adjusting for confounding factors. In contrast, there was no association between antioxidant nutrient intake and AMD among the nonsmokers and former smokers.

**Conclusions:**

These results suggest that increased consumption of fruits and vegetables containing antioxidant components such as vitamin C, α-carotene, and β-carotene may have a protective effect on AMD. These effects may be more evident among current smokers.

## Background

Age-related macular degeneration (AMD) is one of the major causes of irreversible blindness in older adults [1]. Several studies have identified risk factors for AMD, including age [2], family history [3], diabetes mellitus [4], alcohol consumption [5], cigarette smoking [6, 7] and dietary factors [8–11].

Smoking has been reported to be the strongest environmental risk factor for AMD [7, 12]. Toxins in cigarette smoke induce cellular oxidative damage secondary to dysfunction of the biological systems that detoxify reactive oxygen species (ROS) [13] and depletion of circulating antioxidants [14]. In particular, oxidative stress in the retinal pigment epithelium (RPE) is a major contributing factor in the etiology of AMD [15].

Among dietary factors, fruits and vegetables [16], dairy products [17], fish [18], n-3 fatty acid [19], antioxidant vitamins [16, 19, 20], and carotenoids, particularly β-carotene [19], lutein and zeaxanthin [21–23], are protective against AMD. Antioxidant vitamins protect cells from oxidative stress [24]. Carotenoids accumulate in the macular pigment and protect RPE cells from damage [25].

Most previous studies on the associations between diet and AMD have been conducted in Western countries, including America [26, 27], Europe [28, 29], and Australia [8, 9]. In Asia, several studies have been conducted in Japan [19, 30], China [31], and India [32]. Korea is expected to see a substantial increase in the number of elderly people in the next few decades and it has been estimated that the proportion of the population aged ≥ 60 years will be 41.5% by 2050 [33]. Therefore, age-related health complications are becoming more important. According to the Korea National Health Examination Survey (KNHANES) report, the prevalence of smoking among Korean men was 39.3% in 2015 [34]. In addition, Korea has the third highest number of smokers among the organization for economic cooperation and development (OECD) countries [35]. Knowledge on the epidemiology of AMD is essential to meeting future demands for eye health care and support for persons with AMD. Several studies have been carried out in an attempt to investigate the risk factors for AMD in the Korean population [2, 36]. However, the association between diet and AMD has never been studied.

Therefore, the aim of this study was to determine the relationship between F&V and antioxidant nutrient intake including carotenoids and AMD by cigarette smoking status in elderly men, using data from the Korea National Health and Nutrition Examination Survey (2010–2012).

## Methods

### Data source and study population

This study was based on data from the fifth KNHANES (2010–2012), a cross-sectional, nationally representative survey carried out by the Korea Centers for Disease Control and Prevention. The KNHANES uses a stratified, multistage sampling method and consists of a health interview survey, a health examination survey, and a nutrition survey. The response rates for each survey were 81.9, 80.4, and 80.0% in 2010–2012, respectively. This study was approved by the Institutional Review Board of the Korea Centers for Disease Control and Prevention (2010-02CON-21-C, 2011-02CON-06-C, 2012-01EXP-01-2C) and written informed consent was obtained from all subjects. Detailed information about the survey is available on the website (http://knhanes.cdc.go.kr) [37].

The study population comprised elderly men aged ≥ 65 years who responded to the 2010–2012 KNHANES (*n* = 2031). We excluded subjects without 24 h dietary recall data (*n* = 122); those without fundus photograph data (*n* = 439); and those lacking smoking status data (*n* = 56). A total of 1414 participants were included in the final analysis. General characteristics of the study subjects were not significantly different between those included and excluded from the study (data not shown).

### Assessment of AMD

AMD was defined as per a diagnosis by an ophthalmologist in the health examination survey. The outcome used in this study was the presence of any AMD in at least 1 eye. The presence of early- and late-onset AMD was determined on the basis of the fundus photograph [38, 39]. All fundus photographs were graded twice using the International Age related Maculopathy Epidemiological Study Group grading system [40]. Preliminary grading was performed on the site by ophthalmologist. Detailed grading was later done by nine retina specialists experienced in grading early and late AMD. The decision on any inconsistencies between the preliminary and detailed grading was done by another reading specialist. When the fundus photograph for a participant’s eyes were different in severity, the grade was defined based on the more advanced grade. When the fundus photograph for only one eye was available to be assessed, the grade was evaluated by that eye. Early AMD was defined by the following criteria: the presence of soft, indistinct drusen, or reticular drusen, and the presence of distinct drusen with pigmentary abnormalities in the absence of signs of late AMD. Participants were diagnosed with late AMD if they had neovascular AMD or geographic atrophy. Neovascular AMD was defined by either the detachment of the retinal pigment epithelium (RPE) or neurosensory retina, or the presence of hemorrhages in the sub-RPE or subretinal spaces. Geographic atrophy was identified by the presence of a discrete, circular depigmented area ≥ 175 μm in choroidal vessel diameter.

### General characteristics and smoking behavior

The health interview survey and health examination survey were used to obtain the socio-demographic and lifestyle characteristics of the participants, such as age, body mass index (BMI), residential area, education, family income, alcohol consumption, dietary supplement use, and smoking behavior.

BMI was calculated as weight divided by height squared (kg/m^2^). Residential area was categorized as urban or rural. Education level was categorized as less than high school or high school and above. Family income was categorized into 4 groups according to quartile. Alcohol consumption was assessed with 5 categories (never, ≤ 1 drink/mo, 2–4 drinks/mo, 2–3 drinks/wk., or ≥4 drinks/wk. within the last year). Dietary supplement use defined as a binary variable (yes or no), included the supplementation data of the study subjects for longer than 2 weeks during the previous year. Based on smoking status, the study subjects were divided into 3 groups, nonsmokers, former smokers or current smokers.

### Dietary assessment and estimation of dietary intake of carotenoids

We assessed daily dietary intake using data from a single 24-h recall form recorded in the KNAHANES. Participants reported all food and drinks consumed during the previous day in a face-to-face interview.

The food items in this study were categorized into 9 food groups based on other previous study. The intake of foods, energy and 16 nutrients including vitamin and carotenoids were estimated. To estimate carotenoid intake, the carotenoid data was constructed based on the food items of KNHANES and the Carotenoid Content Database from the United States Department of Agriculture. A total of 2247 food items were included in the carotenoid database. The carotenoid database included 72% of all plant foods reported in the 24 h dietary recall method.

### Statistical analysis

All statistical analyses were performed using SAS version 9.4 (SAS Institute, Cary, NC, USA). Distribution differences of the socio-demographic and lifestyle factors of the men grouped by smoking status were analyzed using the PROC SURVEYFREQ procedure. We analyzed the crude weighted mean and standard error of continuous variables by the PROC SURVEYMEAN procedure and statistical differences by smoking status were analyzed with the PROC SURVEYREG procedure. The PROC SURVEYLOGISTIC procedure was used to test our hypothesis about the relationships between fruits and vegetables, antioxidant nutrient intake, and AMD in nonsmokers and smokers. We estimated the odds ratios (ORs) and 95% confidence intervals (CIs) for AMD across the tertiles of fruit and vegetable, vitamin C, vitamin A, and β-carotene intake, where the lowest tertile was set as the reference. Model 1 was adjusted for socio-demographic factors (age, BMI, residential area, education level, and family income). Model 2 was adjusted for the variables in model 1 plus lifestyle factors (alcohol consumption, dietary supplement use, and total energy intake) and history of diseases (diabetes mellitus and hypertension). Further, for former smokers, the data on daily smoking amount and duration of smoking was included in model 2. For the current smokers, current daily smoking amount and duration of smoking were adjusted in model 2.

## Results

### Characteristics of the study population

The subjects’ age, prevalence of AMD, residential area, dietary supplement use, and diabetes mellitus did not differ by smoking status (Table 1). However, the current smokers had lower BMI, educational status and lower family income than the nonsmokers and the former smokers. The ratio of subjects who had higher intake of alcohol was higher among current smokers than the nonsmokers and the former smokers. The ratio of subjects with hypertension was higher among former smokers than the nonsmokers and the current smokers. The subjects included in this study did not differ from excluded subjects in terms of their general characteristics (data not shown).

**Table 1:** Characteristics of participants by smoking status^a^

Variables	Nonsmokers (*n* = 227)	Former smokers (*n* = 856)	Current smokers (*n* = 331)	*p*-value
Age (y)	71.6 ± 0.4	72.1 ± 0.2	71.3 ± 0.3	0.1433
BMI (kg/m^2^)	23.6 ± 0.2	23.4 ± 0.1	22.7 ± 0.2	0.0009
AMD	35 (12.5)	115 (14.5)	56 (18.0)	0.3052
Residential area				
Urban	154 (66.0)	588 (67.5)	207 (62.3)	0.3489
Rural	73 (34.0)	268 (32.5)	124 (37.7)	
Education				
Less than high school	127 (57.3)	509 (64.0)	241 (74.9)	0.0005
High school and above	99 (42.7)	347 (36.0)	90 (25.1)	
Family income				
< 25th	88 (36.1)	373 (44.4)	182 (54.1)	0.0113
25th to 50th	76 (38.2)	251 (29.9)	76 (22.7)	
50th to 75th	38 (16.3)	129 (13.8)	40 (13.4)	
75th to 100th	24 (9.4)	96 (11.8)	30 (9.8)	
Alcohol consumption				
Never	88 (40.2)	281 (32.1)	83 (25.1)	0.0007
≤ 1 mo	41 (19.1)	162 (19.6)	51 (15.6)	
2–4/mo	42 (17.6)	138 (15.1)	52 (16.0)	
2–3/wk	35 (14.4)	124 (14.6)	56 (16.6)	
≥ 4 wk	20 (8.7)	148 (18.7)	89 (26.7)	
Dietary supplement use				
Yes	89 (36.1)	373 (42.2)	113 (34.4)	0.0940
Diabetes mellitus	35 (15.0)	162 (18.2)	68 (19.3)	0.5096
Hypertension	98 (42.2)	438 (52.4)	129 (40.2)	0.0028
Smoking behavior				
Daily smoking amount of former smokers (n/d)	–	29.8 ± 1.2	–	–
Duration of former smoking (y)	–	18.3 ± 0.5	–	–
Daily smoking amount of current smokers (n/d)	–	–	13.8 ± 0.5	–
Duration of smoking (y)	–	–	49.5 ± 0.5	–

Data availability was limited in the following categories: age (*n* = 1414); BMI (*n* = 1414); residential area (*n* = 1414); education (*n* = 1413); family income (*n* = 1403); alcohol consumption (*n* = 1410); dietary supplement use (*n* = 1414); diabetes mellitus (*n* = 1414); hypertension (*n* = 1414); daily smoking amount of former smokers (*n* = 852); duration of former smoking (*n* = 855); daily smoking amount (*n* = 331); duration of smoking (*n* = 331)

*BMI* body mass index, *AMD* age related macular degeneration

^a^Values are mean ± s.e. or n (%); *n* = 1414

The current smokers consumed less food in total, and, in particular, less cereals/potatoes/sugar products, fruits and vegetables than did the nonsmokers and the former smokers (Table 2). Intake of energy, thiamin, vitamin C, vitamin A, and β-carotene were significantly lower in the current smokers than in the nonsmokers and the former smokers.

**Table 2:** Daily foods and nutrients intake by smoking status

	Nonsmokers (*n* = 227)	Former smokers (*n* = 856)	Current smokers (*n* = 331)	*p*-value	
				Model 1^a^	Model 2^b^
Foods					
Total foods (g)	1368.4 ± 62.0	1318.9 ± 31.7	1262.5 ± 47.2	0.2051	0.0049
Cereals/potatoes/sugar products (g)	401.5 ± 14.6	374.3 ± 7.7	358.2 ± 8.8	0.0128	0.0298
Beans/nuts/seeds (g)	41.6 ± 6.2	45.9 ± 3.0	35.6 ± 3.9	0.2132	0.1571
Meats and eggs (g)	70.7 ± 7.4	73.5 ± 5.3	80.9 ± 9.4	0.5601	0.3160
Fishes and shellfishes (g)	39.9 ± 4.9	47.1 ± 3.5	49.5 ± 5.1	0.3195	0.5066
Milk and dairy products (g)	48.3 ± 8.3	49.6 ± 4.7	35.8 ± 6.5	0.5836	0.6781
Fruits and vegetables (g)	578.7 ± 43.4	524.6 ± 19.2	444.0 ± 27.4	0.0020	0.0035
Fruits (g)	204.6 ± 32.8	156.0 ± 12.7	125.2 ± 23.4	0.0570	0.1301
Vegetables (g)	374.1 ± 22.5	368.7 ± 12.4	318.8 ± 12.6	0.0113	0.0062
Mushrooms (g)	4.6 ± 3.1	5.3 ± 1.7	1.5 ± 0.6	0.1014	0.1070
Seaweeds (g)	4.4 ± 1.1	5.1 ± 0.7	5.4 ± 1.9	0.8381	0.8973
Nutrients					
Energy (kcal)	2032.9 ± 67.7	1935.6 ± 36.1	1995.7 ± 47.5	0.5190	<.0001
Carbohydrate (g)	363.9 ± 12.2	340.8 ± 6.7	334.7 ± 7.6	0.0944	0.1450
Protein (g)	66.4 ± 2.5	63.6 ± 1.4	62.3 ± 2.0	0.6056	0.2334
Fat (g)	31.2 ± 2.0	29.0 ± 1.1	27.1 ± 1.4	0.4352	0.2659
Calcium (mg)	480.6 ± 23.9	496.4 ± 14.2	448.0 ± 19.2	0.1844	0.0732
Phosphorus (mg)	1228.6 ± 45.6	1134.1 ± 20.9	1108.8 ± 30.0	0.1332	0.1443
Iron (mg)	17.8 ± 2.0	15.1 ± 0.5	14.4 ± 0.7	0.2512	0.2554
Thiamin (mg)	1.4 ± 0.1	1.2 ± 0.0	1.2 ± 0.0	0.0522	0.0078
Riboflavin (mg)	1.1 ± 0.1	1.1 ± 0.0	1.0 ± 0.0	0.1723	0.1122
Niacin (mg)	15.9 ± 0.5	15.5 ± 0.4	15.3 ± 0.5	0.8700	0.2463
Vitamin C (mg)	105.6 ± 7.1	97.8 ± 3.4	78.7 ± 4.3	0.0003	0.0011
Vitamin A (μgRE)	729.2 ± 54.2	700.6 ± 28.0	601.1 ± 42.0	0.1100	0.0760
α-carotene (mg)	1.0 ± 0.2	0.8 ± 0.1	0.7 ± 0.1	0.1044	0.1323
β-carotene (mg)	4.9 ± 0.4	4.6 ± 0.2	3.6 ± 0.3	0.0148	0.0245
β-cryptoxanthin (mg)	0.6 ± 0.2	0.4 ± 0.0	0.4 ± 0.1	0.6031	0.7048
Lutein + zeaxanthin (mg)	2.9 ± 0.3	2.5 ± 0.2	2.4 ± 0.2	0.3354	0.5297
Lycopene (mg)	1.1 ± 0.3	1.3 ± 0.2	0.7 ± 0.2	0.0695	0.0755

^a^Adjusted for age, BMI, residential area, education level, and family income (*n* = 1401)

^b^Adjusted for age, BMI, residential area, education level, family income, alcohol consumption, dietary supplement use, diabetes mellitus, and hypertension, and total energy intake (*n* = 1397)

### Relationship of F&V and antioxidant nutrient intake and smoking status with AMD

We further evaluated the relationship in separate smoking status (Table 3). As expected, for the current smokers, AMD was inversely associated with F&V [OR (95% CI) = 0.36 (0.14–0.96), p for trend = 0.0576], vitamin C [OR (95% CI) = 0.32 (0.12–0.85), p for trend = 0.0561], α-carotene [OR (95% CI) = 0.23 (0.08–0.67), p for trend = 0.0038] and β-carotene [OR (95% CI) = 0.13 (0.04–0.46), p for trend = 0.0003] intake after adjusted for confounding factors. For nonsmokers and former smokers, however, there was no association between intake of fruits and vegetables and antioxidant nutrients and AMD.

**Table 3:** Odds Ratios (95% CIs) for AMD according to dietary intake in nonsmokers and smokers

	Nonsmokers (*n* = 227)					Former smokers (*n* = 856)					Current smokers (*n* = 331)				
	*n*	AMD	Median	Model 1^a^	Model 2^b^	*n*	AMD	Median	Model 1^a^	Model 2^c^	*n*	AMD	Median	Model 1^a^	Model 2^d^
Fruits and vegetables (g/d)															
Tertile 1	75	8	194.1	1.00	1.00	285	43	195.3	1.00	1.00	110	22	159.6	1.00	1.00
Tertile 2	76	16	481.9	1.93 (0.73–5.12)	2.09 (0.70–6.29)	286	31	443.0	0.69 (0.38–1.25)	0.65 (0.37–1.16)	111	19	366.3	0.79 (0.33–1.86)	0.64 (0.26–1.60)
Tertile 3	76	11	925.1	1.38 (0.43–4.44)	1.28 (0.34–4.82)	285	41	834.2	1.23 (0.64–2.35)	1.17 (0.64–2.15)	110	15	639.1	0.65 (0.25–1.68)	0.36 (0.14–0.96)
*P* for trend				0.6838	0.9960				0.4561	0.4836				0.3844	0.0576
Vitamin C (mg/d)															
Tertile 1	75	11	40.8	1.00	1.00	285	42	34.5	1.00	1.00	110	21	28.5	1.00	1.00
Tertile 2	76	14	84.5	1.65 (0.59–4.63)	1.51 (0.53–4.29)	286	25	75.9	0.66 (0.37–1.19)	0.65 (0.35–1.19)	111	18	64.8	0.49 (0.21–1.14)	0.36 (0.15–0.86)
Tertile 3	76	10	159.8	1.15 (0.35–3.74)	0.76 (0.23–2.58)	285	48	160.6	1.59 (0.88–2.88)	1.57 (0.89–2.75)	110	17	132.6	0.64 (0.27–1.52)	0.32 (0.12–0.85)
*P* for trend				0.9692	0.5177				0.0770	0.0523				0.4130	0.0561
Vitamin A (μgRE/d)															
Tertile 1	75	10	221.0	1.00	1.00	285	44	196.8	1.00	1.00	110	22	156.5	1.00	1.00
Tertile 2	76	12	569.1	1.02 (0.34–3.11)	1.00 (0.31–3.25)	286	36	512.7	0.80 (0.43–1.46)	0.76 (0.42–1.37)	111	16	454.8	0.54 (0.23–1.27)	0.43 (0.17–1.07)
Tertile 3	76	13	1229.3	1.28 (0.44–3.68)	1.05 (0.32–3.41)	285	35	1228.4	0.97 (0.51–1.84)	0.92 (0.50–1.70)	110	18	968.2	0.47 (0.18–1.20)	0.37 (0.13–1.08)
*P* for trend				0.5544	0.8523				0.4956	0.9725				0.0689	0.0740
α-carotene (mg/d)															
Tertile 1	75	10	0.05	1.00	1.00	285	38	0.04	1.00	1.00	110	22	0.04	1.00	1.00
Tertile 2	76	11	0.25	1.38 (0.45–4.18)	1.47 (0.42–5.06)	286	43	0.17	1.24 (0.74–2.09)	1.28 (0.76–2.15)	111	23	0.14	0.65 (0.30–1.39)	0.53 (0.26–1.10)
Tertile 3	76	14	1.51	2.40 (0.81–7.10)	2.20 (0.67–7.21)	285	34	1.28	1.01 (0.53–1.92)	1.07 (0.56–2.03)	110	11	1.15	0.29 (0.12–0.73)	0.23 (0.08–0.67)
*P* for trend				0.1364	0.2745				0.7751	0.8183				0.0038	0.0038
β-carotene (mg/d)															
Tertile 1	75	10	1.30	1.00	1.00	285	46	1.00	1.00	1.00	110	25	0.88	1.00	1.00
Tertile 2	76	14	2.98	2.12 (0.72–6.22)	2.05 (0.69–6.13)	286	34	2.73	0.79 (0.47–1.35)	0.76 (0.45–1.29)	111	16	2.28	0.36 (0.16–0.82)	0.27 (0.11–0.63)
Tertile 3	76	11	7.41	1.36 (0.43–4.28)	1.16 (0.30–4.45)	285	35	7.19	0.94 (0.51–1.74)	0.91 (0.48–1.73)	110	15	5.76	0.24 (0.09–0.62)	0.13 (0.04–0.46)
*P* for trend				0.8311	0.8847				0.9707	0.9439				0.0028	0.0003

*AMD* age related macular degeneration, *BMI* body mass index

^a^Model 1 adjusted for age, BMI, residential area, education level, and family income

^b^Model 2 adjusted for age, BMI, residential area, education level, family income, alcohol consumption, dietary supplement use, and total energy intake

^c^Model 2 adjusted for age, BMI, residential area, education level, family income, alcohol consumption, dietary supplement use, total energy intake, daily smoking amount of former smokers, and duration of former smoking

^d^Model 2 adjusted for age, BMI, residential area, education level, family income, alcohol consumption, dietary supplement use, total energy intake, daily smoking amount of current smokers, and duration of smoking

## Discussion

We found significant inverse associations between F&V and antioxidant nutrient intake and AMD in smokers. The highest tertiles of F&V, vitamin C, α-carotene, and β-carotene intake were associated with significantly reduced odds ratios for AMD compared to the lowest tertiles. In contrast, no statistically significant associations were observed in nonsmokers.

We found that smokers consumed significantly less fruits and vegetables, and antioxidant nutrients, which is consistent with previous studies [41–44]. In this study, F&V intake was 23.3% lower in the current smokers (444.0 g/d) than in the nonsmokers (578.7 g/d). In the Food Habits of Canadians Survey, in adults aged 18–65 years, male smokers showed reduced intake of fruits and vegetables (4.0 servings/d vs. 5.6 servings/d) in comparison with nonsmokers [41]. In a national population-based cohort study conducted in the US, subjects in the highest quartile of fruit and vegetable consumption (29.62 times/week) were more likely to quit smoking and less likely to be heavy smokers than were those in the lowest quartile [42]. The China Seven Cities Study (CSCS) observed that smokers were 46–60% less likely to consume fruit at least once a day than were those who had never smoked [43].

Unlike in other studies [8, 16], no statistically significant associations were observed in nonsmokers and AMD. We found inverse associations between F&V and AMD in current smokers only. Several studies reported that high fruit and vegetable intake is inversely associated with AMD [8, 16]. A cross-sectional study conducted in Australia showed that the proportion of individuals who met the recommended daily intake of vegetables was lower amongst patients with late-stage AMD than in a population of age- and sex-matched controls with no signs of AMD (52.9% vs. 64.5%, *p* = 0.0002) [8]. Recent studies have assessed the associations between healthy dietary patterns, including high fruit and vegetable consumption, and AMD [45, 46]. In the Carotenoids in Age-Related Eye Disease Study (CAREDS), which used the Healthy Eating Index (HEI), subjects whose HEI scores were ranked in the highest quintile (median serving of 3.1 for fruits and 4.6 for vegetables) had a 46% lower risk of early-stage AMD compared to those in the lowest quintile (median serving of 1.5 for fruits and 3.0 for vegetables) [45]. In our study, the mean intake of fruits and vegetables was 578.7 g/d among nonsmokers, which is higher than what is observed in other elderly male populations (Chinese: 313.0 g/d [47], Malaysian: 2.51 svg/d [48], Swedish: 3.3 svg/d [49], American: 3.38 svg/d [50]). Furthermore, the prevalence of hypertension (52.4%), one of the risk factors for AMD, was higher in our study population compared to other study (35.6%) [51]. We presume that in our study, among Korean elderly male nonsmokers other risk factor for AMD will be more meaningful than intake of fruits and vegetables.

We also observed a significant inverse relationship between intake of antioxidant nutrients such as vitamin C, α-carotene, and β-carotene and AMD among current smokers. Major dietary sources of β-carotene include green leafy vegetables and yellow fruits and vegetables such as spinach, carrots, pumpkin, and sweet potato [52]. There has been no observational study showing the relationship of dietary intake and smoking with AMD. However, some experimental studies have shown the protective effects of micronutrients derived from fruits and vegetables, such as vitamin A (particularly β-carotene), vitamin C, vitamin E, folic acid, and phenolic compounds, against smoke-induced toxicity, via prevention of lipid peroxidation [53, 54]. Cigarette smoking causes a depletion of intrinsic antioxidant capacity and thus promotes lipid oxidation [53]. Cigarette smoke-induced free radical generation may be the first step in lipid peroxidation in the membrane of LDL particles. That is, lipid peroxidation of LDL may begin after depletion of intrinsic antioxidants such as vitamin E (α-tocopherol) and β-carotene [53]. These nutrients act as antioxidants, as they have the ability to scavenge free radicals and prevent membrane lipid peroxidation [53]. Hininger et al. reported in an intervention study, which involved increased fruit and vegetable consumption for two weeks providing additional 30 mg/day of carotenoids (10 mg β-carotene, 10 mg lycopene and 10 mg lutein) per day, that serum carotene concentrations in smokers are more susceptible to fluctuate compared to nonsmokers from the intervention study [55]. We presume that these mechanisms account for the clearer effect of high F&V consumption and high antioxidant intake among smokers.

We observed the extreme low values in OR for prevalence of AMD across the tertiles of β-carotene intake among smokers. This may be due to the relatively small number of subjects in the smoking-subgroups. A previous cross-sectional population-based study using the data from the National Health and Nutrition Examination Survey (NHANES) also found that subjects in the highest quintile category of carotenoid intake (lutein/zeaxanthin) had a 90% lower risk for AMD compared with those in the lowest quintile category [OR (95% CI) = 0.1 (0.0–0.9)] [56].

In our study, the smokers consumed fewer milk and dairy products and calcium than the nonsmokers. Although we did not observe any association between milk and dairy product intake and AMD, one recent study reported a significant linear trend, over a 15-year period between consumption of dairy foods and the incidence of late AMD [17]. Further research is needed to confirm these results.

The prevalence of AMD did not differ by smoking status in the current study. However, smoking strongly affect the onset and progression of AMD [7, 57]. Coleman et al. reported that, after 15-year of follow-up, the ORs for early-stage AMD among nonsmokers and smokers aged ≥80 years were 1.63 and 5.49, respectively, in comparison to nonsmokers aged < 80 years [58]. Cigarette smoke causes oxidative damage directly and indirectly. Toxins in smoke cause damage directly by generating a large number of free radicals [59] and indirectly by depleting endogenous circulating antioxidants [14].

This study has several limitations. First, the KNHANES is a cross-sectional study, so we cannot explain the causal relationship between dietary nutrient intake and AMD. Second, our dietary data was derived from a single 24-h dietary recall survey, which may provide an inaccurate estimate of normal diet. However, according to the KNHANES report, variations in data from a single day and 2–10 days of 24-h dietary recall were not much different (3.9% for energy, 14.2% for vitamin A, and 7.8% for fiber) [60]. Furthermore, the difference (30.3%) in vitamin A intake between nonsmokers and smokers is much greater than the within-person variation for vitamin A intake. Third, we examined only dietary intake of antioxidant nutrients, but not dietary supplement intake. We analyzed only the intakes of vitamin A and vitamin C from dietary supplements. There was no significant difference for vitamin A and C from dietary supplements according to smoking status (vitamin A: 292.8 ± 161.6 for nonsmokers, 153.2 ± 31.7 for former smokers, and 89.2 ± 41.6 for current smokers; vitamin C: 121.5 ± 32.8 for nonsmokers, 137.2 ± 20.1 for former smokers, and 93.5 ± 29.8 for current smokers). However, we have limited data on nutrient intakes from dietary supplements [515 subjects (36.4%)] and these data were available for 2010 and 2011 years, but not 2012. Therefore, we were unable to estimate nutrient intakes from foods and supplements.

Nevertheless, to the best of our knowledge, this is the first study to find that the AMD prevalence among cigarette smokers is inversely associated with consumption of F&V and antioxidant nutrients. This observation implies that future studies investigating the protective effect of fruit and vegetable consumption on AMD should consider smoking status. We estimated the association between β-carotene intake and AMD. Several studies have observed associations between carotenoids, particularly lutein/zeaxanthin, and AMD [26, 27, 56]. Thus, further studies are needed to elucidate this relationship between antioxidant nutrients and AMD.

## Conclusions

In conclusion, we found that intake of fruits and vegetables, vitamin C, α-carotene, and β-carotene may protect against AMD in elderly male smokers. Future studies are warranted to explore the mechanisms related to the beneficial role of fruits and vegetables and antioxidant nutrients against AMD in smokers. The current results also suggest that public health interventions for elderly smokers should focus on improving dietary habits, including increasing fruit and vegetable consumption, as well as on smoking cessation.

## Abbreviations

AMD: Age-related macular degeneration
CAREDS: Carotenoids in Age-Related Eye Disease Study
CSCS: China Seven Cities Study
F&V: Fruits and vegetables
HEI: Healthy Eating Index
KNHANES: Korea National Health and Nutrition Examination Survey
NHANES: National Health and Nutrition Examination Survey
OECD: Organization for economic cooperation and development
ROS: Reactive oxygen species
RPE: Retinal pigment epithelium
